# Evaluating the impact of the EU Medical Device Regulation (MDR) on mortality and device availability in Germany

**DOI:** 10.1371/journal.pone.0350175

**Published:** 2026-07-02

**Authors:** Afschin Gandjour

**Affiliations:** Frankfurt School of Finance and Management, Frankfurt, Germany; VitalConnect (United States), UNITED STATES OF AMERICA

## Abstract

**Introduction:**

The EU Medical Device Regulation (MDR), effective since May 25, 2017, aims to enhance the safety and quality of medical devices within the EU. This study models the potential net mortality implications of the MDR in Germany under explicit assumptions about safety improvements, market withdrawals, and certification delays.

**Methods:**

A combined top-down and bottom-up approach was employed to evaluate the impact of the EU MDR on mortality in Germany. The top-down approach estimated avoided deaths using the Keyfitz–Vaupel life-table elasticity, while the bottom-up approach quantified mortality reductions attributable to individual MDR-regulated medical devices. Based on the results of both approaches, the analysis also estimated the number of deaths potentially resulting from market withdrawals and certification delays.

**Results:**

Official German reports attribute an average of 17 device-related deaths annually, whereas international estimates suggest the true figure may reach up to 2,500 per year. Market withdrawals and certification delays associated with MDR implementation were modelled as potentially contributing an estimated 163–1,884 additional annual deaths and a one-time mortality burden of 700–4,200 deaths.

**Conclusion:**

Under central assumptions, the MDR is consistent with long-term reductions in device-related mortality, but transition-period certification bottlenecks and withdrawals—especially for niche/pediatric devices—can offset gains in the short to medium term. Targeted mitigation is warranted to secure benefits and limit unintended harms.

## Introduction

The EU Medical Device Regulation (MDR) was adopted on April 5, 2017, and entered into force on May 25, 2017. Its application became mandatory on May 26, 2021, following a transition period [1]. The MDR replaces the previous Medical Device Directives (MDD) and the Active Implantable Medical Device Directive (AIMD). This regulation was designed to enhance the safety and quality of medical devices within the European Union, addressing issues that arose from incidents like the PIP breast implant scandal.

The PIP scandal, which surfaced in 2010, revealed that the French company PIP used industrial-grade silicone instead of medical-grade silicone in their breast implants [2]. This malpractice led to significant health risks, including a higher likelihood of ruptures and potential inflammatory reactions, affecting hundreds of thousands of women globally and prompting widespread recalls and health concerns. Regulatory authorities—including Germany’s Federal Institute for Drugs and Medical Devices (Bundesinstitut für Arzneimittel und Medizinprodukte, BfArM)—responded by recommending the removal of affected implants and initiating health monitoring for impacted patients.

In Germany, concerns about medical device safety predate the MDR and have been substantiated by adverse event data. According to the BfArM [3], between January 2005 and December 2015, 184 product-related deaths were reported. As of the time of writing this manuscript, this remains the most recent comprehensive data available from BfArM on the issue. However, the reporting system was criticized for being prone to manipulation and underreporting, suggesting the true number of related deaths could be significantly higher [4].

The MDR introduces stricter testing procedures and documentation requirements to ensure that only safe and effective products enter the market. It promotes transparency through traceability and more rigorous clinical evidence requirements, aiming for uniform regulation across the EU, which facilitates free trade and may benefit manufacturers that can comply efficiently with the stricter EU-wide requirements. However, there are concerns about unintended negative consequences. The new requirements impose significant administrative burdens and higher costs for testing and certification. Smaller companies may struggle to meet these demands, leading to potential market exits or product withdrawals. The increased complexity and cost might also slow down the introduction of new products, potentially causing shortages and stifling innovation [5]. BVMed [6] estimates that the implementation costs for the medical technology sector in Germany to comply with the MDR will be between 7 and 10 billion euros.

Furthermore, BVMed warned that up to 30% of medical devices could become unavailable because manufacturers might not meet the stringent new requirements in time [7]. However, the extension of the transition periods to the end of 2027 or 2028, depending on the product’s risk class, provides manufacturers with additional time to ensure their products comply with the regulations. Even with the extension, certain restrictions will persist. Devices that pose an unacceptable risk, those that have undergone significant changes since their initial certification, and those already undergoing the MDR certification process will not qualify for the extended deadlines. Additionally, the current “sell-off” provision, which allows manufacturers to continue selling their existing stock of medical devices certified under the previous directives (MDD or AIMD) even after these directives were replaced by the MDR, will be eliminated [8].

This study aims to evaluate the MDR and estimate the number of deaths potentially avoided under assumed MDR-related safety improvements, as well as deaths potentially associated with product withdrawals and certification delays. To assess the latter, the study employs a modeling approach.

## Methods

### Conceptual approach

The counterfactual scenario in this analysis was defined as the continued application of the prior regulatory framework—namely, the MDD and AIMD. All estimates of deaths avoided were interpreted as modelled improvements relative to this historical baseline under the assumptions specified below. Conversely, harms from market withdrawals and certification delays were modelled as potential consequences of regulatory frictions associated with MDR implementation.

The analysis evaluated the potential net mortality implications of the MDR by comparing modelled reductions in device-related mortality associated with enhanced safety requirements with modelled increases in mortality associated with product withdrawals and certification delays. We modelled scenarios in which MDR-related safety improvements reduced device-related mortality by 50% to 100%, with a base case of 75%. This range was chosen to reflect expected improvements in regulatory oversight while allowing for residual risks from clinical use, inherent device limitations, and incomplete adverse-event reporting.

To estimate the impact of product unavailability, the annual number of deaths currently prevented by MDR-regulated medical devices was multiplied by the likelihood and duration of short- or long-term unavailability, while accounting for the characteristics of affected patient populations and the availability of substitutes. Permanent market withdrawals were modelled as a recurrent annual effect, whereas certification delays were modelled as a one-time transition-period effect. Specifically, the number of additional deaths associated with permanent market withdrawals was estimated using the following formula:


$$\begin{array}{c} Additional\;deaths=D\cdot w\cdot \left[\overset{benefit}{\underset{niche}{s}}\cdot \overset{withdrawal}{\underset{niche}{s}}\cdot \left(\text{1}-c_{niche}\right)+\left(\text{1}-\overset{benefit}{\underset{niche}{s}}\right)\cdot \left(\text{1}-\overset{withdrawal}{\underset{niche}{s}}\right)\cdot \left(\text{1}-c_{non}\right)\right] \end{array}$$ (1)

In this expression, D represents the annual number of deaths currently avoided by MDR-regulated devices, w is the overall device withdrawal rate, $\overset{benefit}{\underset{niche}{s}}\backslash )$ is the share of health benefit accruing to niche or vulnerable populations, and $\overset{withdrawal}{\underset{niche}{s}}\backslash )$ is the share of withdrawn products classified as niche. The parameters $c_{niche}\backslash )$ and $c_{non}\backslash )$ denote the substitution rates for withdrawn niche and non-niche products, respectively. This structure allows the analysis to reflect that niche products are both more likely to be withdrawn and less likely to be replaced, thus disproportionately affecting vulnerable subpopulations.

Certification delays were estimated using the same general unavailability logic but were treated as temporary rather than permanent. The one-time mortality burden from certification delays was calculated by multiplying the annual mortality benefit of MDR-regulated devices by the assumed duration of delay and by the mortality-weighted share of devices temporarily unavailable during the MDR transition period. The mortality-weighted share was derived by combining the estimated share of affected certificates with the estimated share of mortality benefit represented by delayed or temporarily unavailable devices. This approach produced a one-time estimate rather than a recurring annual effect.

Two distinct methodologies—a top-down and a bottom-up approach—were employed to comprehensively assess the mortality benefits of MDR-regulated medical devices over the past two decades. These approaches were used to estimate the annual mortality benefit of MDR-regulated medical devices. This estimate served as the key input D in the market-withdrawal formula and as the baseline annual mortality-benefit parameter in the certification-delay calculation.

### Top-down approach

To estimate the mortality reduction attributable to MDR-regulated medical devices and surgical technologies, we employed a top-down approach based on observed gains in life expectancy and their attributable causes. The analysis began with historical increases in life expectancy in Germany over the past two decades, drawing from official life-table data. To isolate the share of this gain attributable to healthcare delivery and medical technology—versus broader public health or socio-economic improvements—we followed the attribution logic used in previous macro-level assessments of mortality change.

To translate life expectancy improvements into reductions in mortality, we applied the Keyfitz–Vaupel elasticity [9], which approximates the percentage decline in age-specific mortality rates required to produce a unit increase in life expectancy. The resulting estimates were then applied to recent all-cause mortality totals to quantify the number of deaths postponed or avoided annually.

To apportion this aggregate benefit to surgical procedures and medical devices specifically, we relied on two published attribution models. The first was a physician survey study [10] that assessed the contribution of eight major condition groups to mortality improvement in the United States (U.S.) and estimated the relative share attributable to surgical and device-based interventions. The second source was a decomposition analysis of life-expectancy improvements in the U.S. [11], which quantified the share attributable to non-pharmaceutical medical care. We conservatively assumed that surgical and device interventions accounted for one-half of this broader medical contribution. Together, these sources defined the uncertainty bounds for the estimated range of deaths avoided by medical devices and surgical technologies.

### Bottom-up approach

The bottom-up approach estimated the annual number of deaths avoided in Germany by selected MDR-regulated medical device categories. This approach was used as the primary device-specific estimate and was compared with the top-down estimate as an external plausibility check; the two estimates were not summed.

The analysis proceeded in five steps. First, device categories were selected to represent the major MDR-regulated medical technologies with potential relevance for mortality in Germany, rather than only those for which favourable mortality evidence was available. Categories were considered if they fell within the scope of the EU MDR and were used in patient populations with non-negligible mortality risk. For each category, we then identified available registry, epidemiological, and clinical evidence to estimate the treated population and the incremental mortality effect. If evidence was insufficient to support a clear incremental all-cause mortality benefit, the category was retained in the analysis but assigned a zero mortality benefit in the base case. The included categories were diagnostic imaging devices, implantable cardioverter-defibrillators, cardiac resynchronisation therapy devices, dialysis machines, minimally invasive and robotic surgical technologies, continuous glucose monitors, blood-pressure monitors, transcatheter heart-valve technologies, ventilators, insulin pumps, external defibrillators, prosthetics and orthotics, and advanced wound-care products.

Second, the annual number of patients exposed to or treated with each device category was estimated. German data sources were used wherever available, including national registries, quality-assurance reports, statutory health-insurance data, professional society reports, and German epidemiological studies. For continuously used technologies, such as implantable defibrillators, insulin pumps, continuous glucose monitors, dialysis machines, and blood pressure monitors, prevalent users were used as the relevant annual patient population. For procedure-based technologies, annual procedure or treatment volumes were used. Where German data were unavailable, European or international estimates were extrapolated to Germany and labelled as such.

Third, mortality-effect estimates were identified from clinical trials, meta-analyses, registry studies, and epidemiological studies. Evidence was prioritized in the following order: randomized controlled trials or meta-analyses reporting mortality outcomes; large contemporary registries reporting survival; German or European studies; and, where necessary, international studies considered transferable to the German setting. Because this was not a systematic review, we did not conduct a formal database search with predefined search strings. Instead, we used a targeted evidence-identification strategy, prioritizing randomized trials, meta-analyses, large registries, and German or European data directly reporting mortality or survival. Where multiple estimates were available, the estimate most closely matching the German target population and comparator was selected; where evidence was indirect, conservative assumptions were used.

Fourth, the analysis estimated the incremental mortality benefit of contemporary or advanced MDR-regulated devices relative to a clinically relevant comparator. Depending on the device category, the comparator was earlier-generation technology, standard non-device treatment, conventional surgery, usual care, or delayed/no access to a clinically equivalent substitute. The analysis therefore did not attribute all survival among treated patients to the device, but only the incremental mortality reduction relative to the comparator. To ensure consistency with the top-down approach, the comparator generally reflected technologies or care pathways available approximately two decades ago.

Fifth, deaths avoided were calculated by multiplying the annual patient population by the estimated absolute annual mortality reduction:


$$\begin{array}{c} {\text{Deaths avoided}}_{i}=N_{i}\times ARR_{i}, \end{array}$$ (2)

where $N_{i}$ is the annual number of patients treated with device category i, and $ARR_{i}$ is the absolute annual mortality reduction attributable to that device category. When studies reported relative mortality reductions, these were converted into absolute annual mortality reductions by applying the relative effect to the baseline annual mortality risk of the relevant patient population. When studies reported multi-year survival, annual mortality rates were derived assuming a constant hazard over the reported follow-up period.

Device-specific estimates were then summed across categories to obtain the total bottom-up estimate. Potential overlap between categories was addressed by excluding duplicated patient groups where possible. For example, CRT-D patients were not counted separately in the CRT-P category because they were already included in the ICD population.

### Reconciliation of top-down and bottom-up estimates

The top-down and bottom-up approaches were used for complementary but distinct purposes. The bottom-up approach provided a device-specific estimate of the annual mortality benefit of selected MDR-regulated medical devices, yielding approximately 16,500 deaths avoided annually. However, this subtotal likely underestimates the full mortality benefit of MDR-regulated devices because several cardiovascular and neurovascular technologies were not included. The top-down approach therefore served as an independent aggregate plausibility range, estimating 13,000–25,000 deaths avoided annually based on life-expectancy gains and published attribution estimates for surgical and device-related innovations. The two estimates were not added. Because the bottom-up estimate fell within the top-down range but was likely incomplete, the midpoint of the top-down range—19,000 deaths avoided annually—was used as the central aggregate input in downstream analyses, with 13,000 and 25,000 used as lower and upper bounds. Using 19,000 rather than the lower bottom-up subtotal is conservative for estimating access-related harms, because it reduces the risk of understating the mortality consequences of device unavailability.

### Sensitivity analysis

To explore the robustness of the findings and assess the conditions under which the MDR may produce a net mortality benefit or harm, two forms of sensitivity analysis were conducted. First, a Monte Carlo simulation (MCS) was performed to account for uncertainty in the two most influential parameters: the annual number of deaths potentially averted through MDR-related safety improvements, allowing for the fact that some device-related mortality may persist after implementation, and the number of additional deaths potentially associated with permanent product withdrawals. Both parameters were modeled using triangular distributions based on minimum, most likely, and maximum values derived from published estimates and expert assessments. In each of 1,000 iterations, random draws from these distributions were used to calculate the net annual mortality impact of the MDR. The proportion of iterations yielding a negative result—i.e., where added harms outweighed safety benefits—was recorded to estimate the probability of net harm.

In addition, a threshold analysis was carried out to determine which parameter values would lead to a breakeven point, defined as zero net mortality impact. Specifically, we identified the input values that would produce an equal number of deaths averted and added annually. This approach allowed us to quantify the tipping points at which the MDR’s benefits no longer offset its unintended consequences. These analyses jointly provide insight into the robustness of the base-case conclusion and the relative importance of individual assumptions.

## Results

### Health benefits of MDR

According to the German Federal Institute for Drugs and Medical Devices (BfArM), a total of 184 deaths were reported between January 1, 2005, and December 31, 2015, as being associated with medical device incidents (BfArM, 2016). This equates to an average of approximately 17 deaths per year, based on spontaneous reports. However, this figure likely underrepresents the true burden of device-related mortality due to underreporting, definitional ambiguity, and relatively lenient reporting requirements prior to the MDR [4]. In support of this, Craig et al. [12] estimate that in Australia only about 0.5% of adverse device events are officially reported. If this rate applied to fatal cases in Germany, the actual number of deaths could exceed 3,000 annually—although this likely overstates the true burden, as fatal events are more frequently reported than non-fatal ones.

In the United States, the Food and Drug Administration’s (FDA) Manufacturer and User Facility Device Experience (MAUDE) database recorded approximately 83,000 death-associated reports from 2008 to 2017, equating to an estimated 8,000–10,000 deaths annually [13]. Moreover, a recent analysis found that nearly one-quarter of device-related deaths in the MAUDE database were misclassified under other categories, such as injury, malfunction, or “other” [14]. Accounting for this underreporting implies that the true number of annual U.S. device-related deaths could exceed 10,000. Adjusted for Germany’s population (approximately 84 million compared to 331 million in the U.S.), this would correspond to an estimated 2,500–3,000 deaths per year in Germany.

Further support for a higher estimate comes from regulatory comparisons. Van Norman [15] noted that unlike the FDA, the pre-MDR EU regulatory framework did not require clinical efficacy data for high-risk or first-in-class devices. Hwang et al. [16] similarly reported that devices approved first in the EU were 2.9 times more likely to be subject to post-market safety alerts or recalls than those first approved in the U.S. These findings imply that riskier products were more likely to reach the European market prior to MDR implementation, reinforcing the plausibility—or even conservative nature—of the 2,500–3,000-death upper bound for Germany.

As a third reference point, media-reported data from the UK’s Medicines and Healthcare products Regulatory Agency (MHRA) suggest approximately 335 device-related deaths annually [17]. Scaled to Germany’s population, this translates to an estimated 420 deaths per year.

Taking these benchmarks together, a conservative and reasonable estimate for true device-related mortality in Germany likely falls between 300 and 2,500 deaths annually. The lower bound is set above the official German spontaneous-reporting figure to account for underreporting while remaining close to the population-scaled UK estimate. The upper bound reflects population-adjusted extrapolation from U.S. MAUDE data and is intended to capture a plausible high-burden scenario. Based on this range, and as outlined in the methods section, we assume that the MDR reduces this mortality burden by 50% to 100%, with a base case of 75% to reflect both expected improvements and residual challenges in device oversight, performance, and reporting. Under these assumptions, this corresponds to a modelled annual reduction in device-related deaths ranging from 150 to 2,500, with the base case yielding a reduction of approximately 225–1,875 deaths per year.

### Harm by MDR

#### Top-down approach.

Germany gained approximately two years in life expectancy over the past two decades [18]. Previous analyses suggest that about half of this gain—roughly one year—is attributable to improvements in healthcare delivery and medical technology, while the other half reflects broader public-health and socio-economic factors such as better nutrition, reduced smoking, and increased physical activity [19].

Using the Keyfitz–Vaupel elasticity, a one-year gain in life expectancy implies a sustained 9.5% reduction in age-specific mortality rates [9]. Applied to Germany’s 1,020,000 deaths in 2023 [20], this translates to approximately 97,000 deaths avoided per year for each additional year of life expectancy—corresponding to about 195,000 deaths avoided annually for a two-year gain.

To isolate the contribution of surgical procedures and medical devices, we relied on attribution data from Wamble et al. [10], who estimated that these two categories together account for 25% (95% CI: 22%–28%) of mortality improvements across eight leading disease categories in the U.S. Applying this proportion to the 97,000 deaths avoided suggests that approximately 25,000 (95% CI: 22,000–28,000) deaths are prevented annually due to surgical and device-based innovations.

As a conservative cross-check, we used estimates from Buxbaum et al. [11], who found that 13% of the gain in U.S. life expectancy between 1990 and 2015 was attributable to medical non-pharmaceutical care. Assuming that surgical procedures and medical devices account for half of this category yields an attributable share of 6.5%. Applying this share to the approximately 195,000 deaths avoided annually implied by Germany’s two-year gain in life expectancy gives 12,675 deaths avoided annually, rounded to approximately 13,000.

Taken together, these estimates suggest that 13,000–25,000 deaths are avoided annually in Germany as a result of surgical procedures and MDR-regulated medical devices.

#### Bottom-up approach.

The bottom-up approach estimated the annual number of deaths avoided by MDR-regulated medical devices in Germany by assessing the contributions of selected device categories. The estimate of approximately 16,500 deaths avoided annually (Table 1) includes diagnostic imaging machines, implantable defibrillators, dialysis machines, minimally invasive surgical tools, robotic surgery systems, continuous glucose monitors, blood-pressure monitors, artificial heart valves, ventilators, insulin pumps, external defibrillators, and advanced wound-care products. Each device category’s impact was estimated as the incremental mortality benefit of contemporary or advanced device-based care relative to a prespecified comparator, such as earlier-generation technology, standard non-device care, conventional surgery, usual care, or no clinically equivalent substitute. Where applicable, these comparators were chosen to reflect technologies or care pathways available approximately two decades ago, consistent with the time horizon used in the top-down analysis. The following section provides detailed information on the eligible patient populations, comparators, and corresponding mortality reductions for each device category.

**Table 1:** Estimated impact of MDR-regulated medical devices on annual deaths avoided in Germany. See Results section for further information and discussion.

Medical device category	Comparator/alternative treatment	Annual patient number	Absolute mortality reduction per year	Deaths avoided annually	References
Diagnostic imaging	Management without modern CT/MRI	26,600,000	0%	0	[21]
Implantable defibrillators	First-generation ICD therapy	190,698	1.5%	2,765	[22; 23; 24; 25]
CRT-P	First-generation CRT-P therapy	33,000	5%	1,518	[26, 27]
Prosthetics and orthotics	Earlier-generation prosthetic/orthotic care or no adequate device-supported rehabilitation	62,016	2.0%	1,240	[28]
Dialysis machines	Standard high-flux hemodialysis	87,255	2.0%	1,745	[29; 30]
Minimally invasive surgery	Conventional open surgery	1,500,000	0.1%	1,500	[31]
Robotic surgery	Conventional open or laparoscopic surgery	50,000	0.1%	50	[32; 33]
Continuous glucose monitors	Conventional self-monitoring of blood glucose without CGM	500,000	0.1%	500	[34]
Blood pressure monitors	Usual care without home blood-pressure monitoring	8,750,000	0.03%	2,625	[35,36,37]
TAVI	Surgical aortic valve replacement or conservative medical management	100,817	0.1%	101	[38]
Ventilators	Conventional/less advanced ventilatory support	265,573	1.0%	2,656	[39]
Insulin pumps	Traditional open-loop CSII or multiple daily injections	200,000	0%	0	[40]
External defibrillators	Historical OHCA care with lower AED availability	24,872	7.0%	1,658	[41, 42]
Advanced wound care products	Conventional wound care	370,000	0.05%	185	[43; 44]

For dialysis technologies, the comparator was standard high-flux hemodialysis. Based on Blankestijn et al. [30], high-dose hemodiafiltration was associated with lower mortality than conventional high-flux hemodialysis. We approximated the incremental mortality effect of advanced dialysis technology as an absolute annual reduction of 2 percentage points. This assumption was applied to the estimated German dialysis population.

For robotic surgery, the comparator was conventional open or laparoscopic surgery. Because mortality rates for robot-assisted surgery are generally comparable to those of traditional open or laparoscopic approaches, only a minimal incremental mortality benefit was assumed in the base case [33]. This assumption reflects the limited evidence for a consistent all-cause mortality advantage, although some studies have reported mortality benefits for selected robotic procedures [32]. Similarly, open and laparoscopic surgeries exhibit largely similar mortality rates, though some studies underscore the advantages of laparoscopic techniques [31].

Three major life-saving device categories at the population level are implantable cardioverter-defibrillators (ICDs), cardiac resynchronization therapy (CRT) systems, and external defibrillators. For ICDs, a modeling study estimated the prevalence in Germany to be 190,698 in 2022 [24]. To isolate the incremental survival gain from contemporary ICD technology, we compared modern-era mortality rates with those achieved by first-generation devices. A recent cohort reported a 5-year survival of 73% among ICD recipients [25], corresponding to an annual mortality of ~6.1% (1 - 0.73^1/5). In contrast, annual mortality in the ICD arms of first-generation trials was ~ 6.3% in SCD-HeFT ([22]; 22% deaths over 45.5 months) and ~8.8% in MADIT II ([23]; 14.2% deaths over 20 months). Taken together, these figures imply an incremental reduction in annual mortality of 0.23–2.68 percentage points for contemporary ICD therapy; using the average of the two trials as a base case yields a 1.45-percentage-point reduction (7.55% → 6.10%). Applied to an estimated 190,698 ICD users in Germany, this corresponds to approximately 2,770 additional lives saved per year (range ~440–5,100).

For CRT systems, the comparator was first-generation CRT-P therapy, rather than no resynchronisation therapy. To estimate the incremental mortality benefit of current CRT systems, we first derived contemporary and historical annual death rates. The Institut für Qualitätssicherung und Transparenz im Gesundheitswesen (IQTiG) device audit [45] reported a 36-month Kaplan–Meier survival of 89% for German patients implanted with CRT devices between 2018 and 2021. Assuming a constant hazard, this corresponds to an annual mortality rate of 3.9% $\left(\text{1}-{\text{0}.\text{8}\text{9}}^{\text{1}/\text{3}}\right)$. In contrast, the first-generation CARE-HF trial observed a lower survival rate of 80% over a median of 29.4 months among CRT-P recipients [26], implying an annual mortality rate of approximately 8.5% $\left(\text{1}-{\text{0}.\text{8}\text{0}}^{\text{1}/\text{2}.\text{4}\text{5}}\right)$. The resulting difference—approximately 4.6 percentage points per year—was interpreted as the incremental mortality benefit of contemporary CRT-P systems relative to first-generation CRT-P therapy, reflecting advances in lead technology, device programming, patient selection, follow-up care, and remote monitoring.

Prevalence of CRTs was derived from the same IQTiG dataset (2023), which documented 68,393 unique CRT patients with at least one billed device check in 2020. Adjusting for an estimated 5–6% of non-attenders yields approximately 72,000 CRT users in 2022. Since CRT-D devices are already included in the national ICD stock ([24], counting “Einkammer- und Zweikammer-ICD sowie CRT-D”), we excluded this overlap by applying the CRT-P share reported in the German Pacemaker & ICD Register—approximately 45% [46]. Applying a 4.6 percentage point absolute risk reduction to approximately 33,000 CRT-P patients results in an estimated 1,500 additional deaths prevented annually, which can be added to the bottom-up tally without double-counting ICD benefits.

For automated external defibrillators (AEDs), the comparator was historical out-of-hospital cardiac arrest care with substantially lower AED availability. Based on an incidence rate of approximately 30 out-of-hospital resuscitations per 100,000 inhabitants [41], the total number of resuscitations in Germany, with a population of approximately 84 million, was estimated at about 25,000 annually. Approximately 9 patients per 100,000 inhabitants are discharged alive from hospital after resuscitation [41], corresponding to a current survival rate of approximately 30% (9/30). Historical survival after out-of-hospital cardiac arrest was assumed to be approximately 10% two decades ago [42]. Because the improvement from 10% to 30% survival likely reflects several factors—including bystander cardiopulmonary resuscitation, emergency medical services, post-resuscitation care, and AED deployment—we attributed one third of the 20-percentage-point survival gain to AED availability. This corresponds to an AED-attributable absolute survival gain of approximately 6.7 percentage points and an estimated 1,700 additional lives saved annually (25,000×0.067).

For CT and MRI, the comparator was diagnostic and clinical management without modern cross-sectional imaging in the relevant indications. Randomized and observational evidence indicates that modern cross-sectional imaging can confer substantial cause-specific survival benefits in selected high-risk populations. For example, low-dose CT screening reduces lung-cancer mortality by 20–26% [47], coronary CT angiography-guided management lowers the composite outcome of coronary death or non-fatal myocardial infarction over long-term follow-up (SCOT-HEART extension; [48]), and contrast-enhanced breast MRI surveillance in BRCA1/2 mutation carriers is associated with a substantial reduction in breast-cancer mortality [49]. However, these mortality benefits apply to selected indications and high-risk groups, which probably account for only a small share of the approximately 27 million CT/MRI examinations performed annually in Germany. For most CT/MRI examinations, direct evidence of an all-cause mortality benefit is limited, and potential marginal gains from earlier diagnosis may be partly offset by the small but non-negligible risk of radiation-induced malignancy associated with CT imaging, though not MRI [50]. Therefore, to avoid extrapolating indication-specific mortality benefits to all diagnostic imaging use, the base-case all-cause mortality effect for CT/MRI was set to zero.

For home blood-pressure monitoring (HBPM), the comparator was usual care without home monitoring. A 65-trial meta-analysis involving 21,053 participants reported that HBPM reduces office systolic/diastolic blood pressure by –3.27/–1.61 mm Hg compared with usual care, even without telemonitoring co-interventions [36]. Because direct mortality evidence for HBPM is limited, the mortality effect was estimated indirectly by translating the observed systolic blood-pressure reduction into an expected mortality reduction. Prior evidence suggests that each 5 mm Hg decrease in systolic blood pressure is associated with a 7% reduction in all-cause mortality [37]. Applying this relationship linearly, a –3.27 mm Hg reduction corresponds to an estimated relative all-cause mortality reduction of approximately 4%. Assuming an annual mortality risk of 1% among treated hypertensive individuals, this translates into an absolute annual mortality reduction of approximately 0.04 percentage points. To account for imperfect adherence and the indirect nature of this conversion, the base-case mortality benefit was rounded down to 0.03 percentage points per year.

Conventional open-loop continuous subcutaneous insulin infusion (CSII) pumps have been part of routine clinical practice since the early 1990s. Although the adoption of hybrid closed-loop or automated insulin delivery (AID) systems has accelerated in Germany, registry analyses and randomized controlled trials have not yet demonstrated a statistically significant advantage in all-cause mortality for AID compared to traditional open-loop CSII [40].

This detailed, device-specific analysis underscores the critical role of MDR-regulated medical technologies in enhancing patient outcomes and extending life expectancy in Germany.

#### Annual deaths from market withdrawals.

Taken together, the top-down and bottom-up approaches suggest that between 13,000 and 25,000 deaths are avoided annually in Germany due to the use of MDR-regulated medical devices. However, as an unintended consequence of MDR implementation, a non-trivial number of products—particularly niche devices designed for small patient populations with limited or no therapeutic alternatives—have been withdrawn from the market. Surveys indicate that 6% to 30% of devices may be affected by certification-related market withdrawals [7, 5].

To estimate the mortality impact of device withdrawals, we distinguish between niche and non-niche medical devices, recognizing differences in both their likelihood of withdrawal and the availability of suitable substitutes. Niche devices—those serving patients with rare diseases, pediatric-specific needs, or other medically complex conditions—are more vulnerable to market exit and are often irreplaceable within their therapeutic category. Between 30% and 65% of discontinued products are classified as niche, with the highest share reported by micro-enterprises [5]. The population most affected by the loss of such devices is approximated using the European Commission’s estimate [51] that 6.7% of the EU population lives with a rare disease. When accounting for additional vulnerable subgroups, such as children requiring pediatric-specific devices or patients with highly individualized therapeutic needs, this share may rise to 10–15%. These populations are more dependent on niche products and are generally less likely to benefit from mainstream substitute devices.

Survey data suggest that, across all product categories, only 36% of discontinued devices are fully compensable by alternatives [5]. This substitution rate is likely lower for niche devices due to their specialized design and smaller target populations. For analytical purposes, we assume a substitution rate of 20% to 36% for niche products and 45% to 70% for non-niche products. In the base-case scenario, we apply a substitution rate of 36% for niche devices and 70% for non-niche devices.

The number of modelled additional deaths associated with market withdrawals is estimated using Eq 1. Based on this approach, three illustrative scenarios are calculated:

- Conservative scenario
  - Parameters: D=13,000, w=6%, $\overset{\text{benefit}}{\underset{\text{niche}}{s}}=\text{6}.\text{7}\%$, $\overset{\text{withdrawal}}{\underset{\text{niche}}{s}}=\text{30}\%$, $c_{\text{niche}}=\text{36}\%$, $c_{\text{non}}=\text{70}\%$
  - Estimated additional deaths: ≈ 163 per year
- Base-case scenario
  - Parameters: D=19,000, w=15%, $\overset{\text{benefit}}{\underset{\text{niche}}{s}}=\text{10}\%$, $\overset{\text{withdrawal}}{\underset{\text{niche}}{s}}=\text{40}\%$, $c_{\text{niche}}=\text{36}\%$, $c_{\text{non}}=\text{70}\%$
  - Estimated additional deaths: ≈ 535 per year
- High-impact scenario
  - Parameters: D=25,000, w=30%, $\overset{\text{benefit}}{\underset{\text{niche}}{s}}=\text{15}\%$, snichewithdrawal=65%, $c_{\text{niche}}=\text{20}\%$, $c_{\text{non}}=4\text{5}\%$
  - Estimated additional deaths: ≈ 1,589 per year

**Fig 1:**
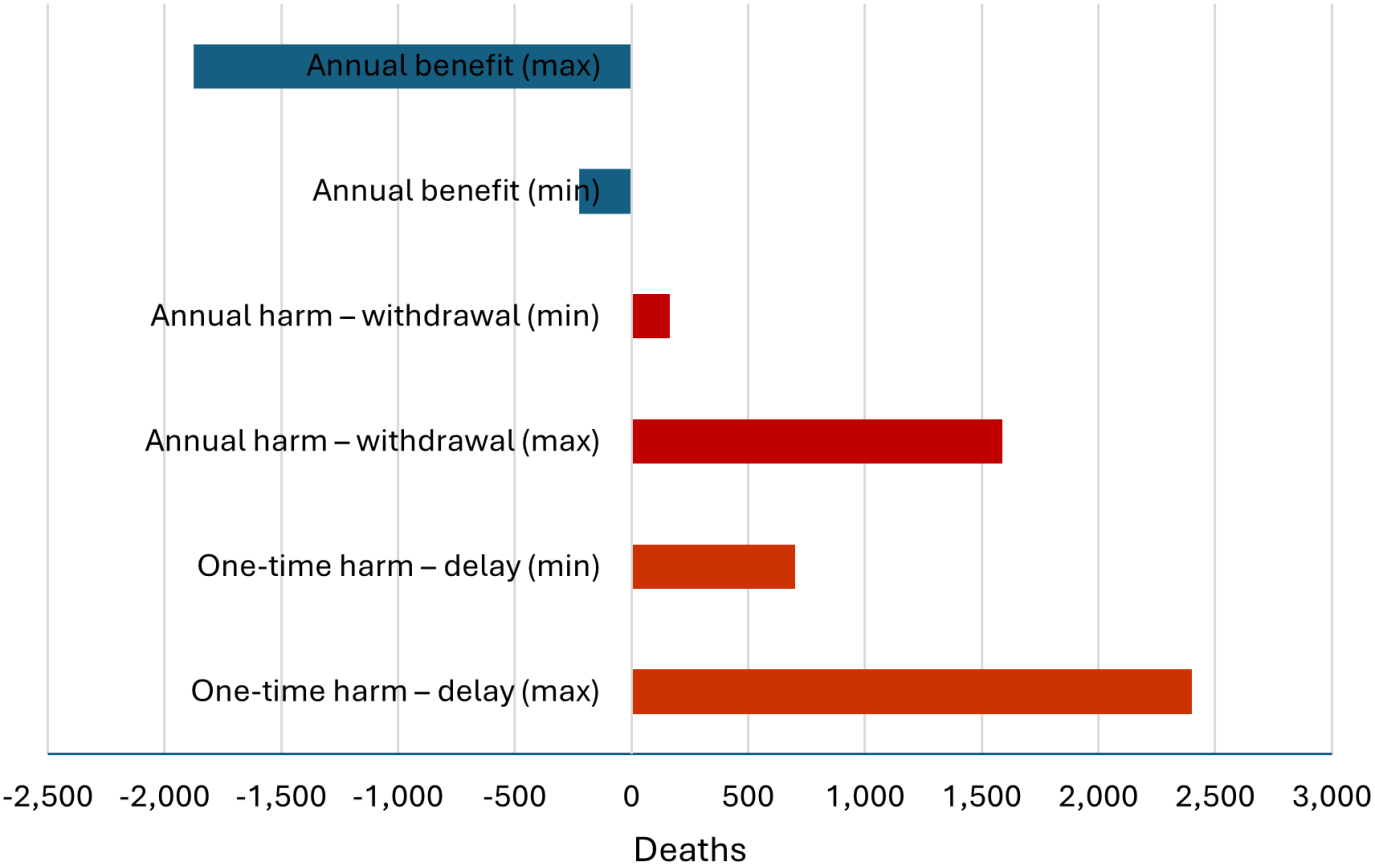
Deaths avoided versus incurred under alternative MDR implementation scenarios. MDR: Medical device regulation.

These estimates underscore the potentially considerable potential mortality burden associated with MDR-related market withdrawals, particularly for vulnerable patients relying on niche medical devices. To illustrate the balance between mortality benefits and harms, Fig 1 presents deaths avoided versus incurred under alternative MDR implementation scenarios.

#### Deaths from certification delays.

Certification delays under the MDR may affect patient outcomes if they temporarily reduce access to critical medical devices. These effects are best understood as a one-time shock concentrated during the MDR transition period. Once certification is completed, affected devices are expected to return to market, restoring availability and stabilizing supply.

According to MedTech Europe [52], the majority of certifications are delayed between 13 and 18 months, with some—particularly innovative or high-risk products—exceeding 24 months. Fewer than 10% of certificates are issued within 6 months. While certification delays affect a large share of devices undergoing MDR conformity assessment, only a subset of these delays results in temporary product unavailability. For legacy devices, whose CE certificates expire before MDR recertification, market access is immediately suspended. According to the European Commission, 3,509 of the 21,376 certificates scheduled to expire between January 2023 and May 2024 had already lapsed as of January 2023, implying that roughly 16% of devices may be unavailable at a given time during the certification bottleneck [53].

Manufacturers, facing resource constraints and the complexity of MDR requirements, tend to prioritize their most commercially and clinically important products. Devices with the highest patient volume, strongest evidence base, or largest installed base—such as ICDs, CRT-P systems, dialysis machines, ventilators, and AEDs—are typically prioritized for early recertification [54]. These high-impact Class III products account for only about 11% of all MDR certificates issued [55], yet they deliver approximately 63% of all mortality benefits associated with medical devices (see Table 1). Consequently, most of the delayed or lapsed certificates concern Class IIa/IIb devices with lower clinical impact.

To reflect this skew, we adjust the mortality impact of device unavailability by applying a composite mortality-weighted factor. Specifically, we multiply the share of devices affected (≈ 16%) by the share of mortality benefit they represent (30%–40%), resulting in an effective mortality-weighted impact range of approximately 5%–6%.

To estimate the number of deaths during the transition period, we model three scenarios varying by delay duration and baseline mortality benefit:

- Minimum Estimate (13 months delay):

13,000 × 1.08 × 5% = 702 deaths

- Best Estimate (15 months delay):

19,000 × 1.25 × 5.6% = 1,330 deaths

- Maximum Estimate (18 months delay):

25,000 × 1.5 × 6.4% = 2,400 deaths

This estimation provides a range for the potential one-time increase in deaths potentially associated with certification delays under the MDR.

#### Sensitivity analysis.

In the sensitivity analysis, the Monte Carlo simulation indicated that the probability of breakeven—i.e., the scenario in which the mortality harms due to product withdrawals equal the mortality benefits of the MDR—was approximately 28%. This indicates that while a net benefit is more likely, there remains a considerable probability of net harm. Threshold analysis further revealed that at least 515 deaths must be averted annually to offset the estimated mortality impact from product unavailability. Similarly, if the benefit of MDR-regulated medical devices in terms of deaths avoided rises to 37,000, or if the product withdrawal rate reaches 29%, the resulting mortality harm would equal the estimated benefit.

## Discussion

The total number of deaths avoided annually by MDR-regulated medical products—estimated at approximately 16,500 using a bottom-up approach—aligns with two independent published estimates, lending face validity to the magnitude of overall device benefits. When isolating the MDR’s net mortality effect, however, the uncertainty intervals for harms from permanent withdrawals (≈163–1,589 additional deaths/year) and for safety gains from enhanced oversight (≈225–1,875 deaths averted/year) substantially overlap. On the basis of ranges alone, the sign of the net effect is therefore indeterminate.

Using our central specification, the model is consistent with a modest net benefit (modelled safety gains exceed modelled withdrawal-related harms). This difference is driven by base-case assumptions—moderate withdrawal rates with partial substitution for non-niche products, a mid-range niche share among withdrawals, and a 75% safety-improvement effect—each chosen as a midpoint of empirically grounded ranges. This central advantage is potentially justified if certification bottlenecks ease over time, if withdrawn non-niche products are largely substitutable, and if MDR post-market surveillance materially reduces device-related mortality. Conversely, it may be not justified if withdrawals remain concentrated in niche or pediatric indications with low substitutability or if the safety effect is smaller than assumed. Consistent with this ambiguity, our Monte Carlo analysis indicates a 28% probability that mortality harms could exceed benefits—highlighting that while a net gain is plausible under central assumptions, a non-trivial risk of net harm remains.

One-time harms from certification delays during the MDR transition are estimated at 700–2,400 additional deaths, with magnitude driven by the length of delays and the share of affected products. Because these effects are front-loaded, they can temporarily offset annual safety gains, yielding an ambiguous net effect in the short term. The net turns positive once recertification restores availability and cumulative safety gains exceed transition losses; the timing of this breakeven depends on recertification throughput, the mix of niche versus non-niche products, and the availability of substitutes. While long-run benefits plausibly outweigh steady-state harms, the near-term net impact remains uncertain due to these transition effects.

Although this study quantifies mortality effects specifically for Germany, the underlying mechanisms of the EU MDR—particularly increased certification requirements and market disruptions—are applicable across all EU member states [52]. However, absolute figures are likely to vary based on national patterns of device usage, healthcare system structures, and the specifics of regulatory implementation. Future studies should replicate this approach in other EU contexts to assess generalizability and to inform a cohesive, pan-European regulatory strategy.

Cross-national comparisons also highlight the tension between safety and access. A U.S. cross-sectional study of 156 Class I-recalled medical devices [56] revealed that 44.1% had been cleared via the FDA’s 510(k) pathway using predicates with prior recall history. Moreover, 48.1% of the devices later served as predicates for other devices that were also recalled. Devices cleared using predicates with prior Class I recalls had a 6.4-fold higher risk of subsequent Class I recall. These findings underscore the need for more robust premarket safeguards in the U.S. system. While the EU-MDR’s more rigorous requirements aim to prevent such safety lapses, they may inadvertently contribute to market access delays or product withdrawals, potentially diminishing some of their intended public health benefits. These contrasting regulatory philosophies highlight different risk-benefit trade-offs.

While this study focuses on mortality as a primary outcome, the MDR’s impact extends well beyond this endpoint. Important secondary outcomes include non-fatal adverse events, patient quality of life, healthcare costs, and the pace of innovation. These broader consequences—both positive and negative—are critical to consider in future evaluations to provide a more complete picture of the MDR’s public health and economic implications.

In addition to these health-related effects, the MDR may pose substantial economic challenges, especially for small and medium-sized enterprises (SMEs). Increased regulatory complexity and certification costs risk triggering product discontinuations, job losses, and a slowdown in device innovation—consequences that remain difficult to quantify fully but may indirectly affect patient access and care outcomes.

The conflict between the need to enforce strict regulations to ensure the safety and efficacy of medical devices and the economic pressure on manufacturers to remain profitable is complex. Several solutions can be considered to resolve this conflict. Continuous dialogue between regulatory authorities, manufacturers, and other stakeholders is crucial to finding a balance between safety and market viability. SMEs could receive financial aid in the form of subsidies or low-interest loans to cover the costs of (re-)certification. Tax incentives could be introduced to alleviate the financial burden on manufacturers arising from compliance with new regulations. Expanding the number and capacity of notified bodies could help avoid bottlenecks and accelerate certification processes. Implementing standardized certification processes could enhance efficiency and reduce costs for manufacturers. Regulations could adopt a risk-based approach, applying the strictest requirements only to products that pose a higher risk to patients. Simplified (re-)certification processes could be introduced for products that have been proven safe over a long period. Leveraging digital technologies and artificial intelligence could improve the efficiency of certification processes and reduce costs.

To address the regulatory challenges posed by the EU MDR, potential solutions can be informed by experiences from other regulatory systems and industries. In Germany, the Federal Institute for Drugs and Medical Devices (BfArM) offers scientific advice to manufacturers, allowing for early dialogue to reduce regulatory uncertainty. However, uptake—particularly among SMEs—may be constrained by factors such as limited internal resources or uncertainty about the added value. Japan’s Pharmaceuticals and Medical Devices Agency (PMDA) has institutionalized early engagement more systematically and integrates it closely into regulatory review timelines, potentially improving predictability and alignment [57]. In the United States, the FDA’s Breakthrough Devices Program provides structured mechanisms to accelerate access to high-impact innovations while aiming to preserve safety standards [58]; at the same time, known limitations of FDA’s predicate-based 510(k) pathway underscore the need for strong pre- and post-market safeguards. Outside of healthcare, the aviation industry employs performance-based regulation that emphasizes outcome-focused oversight, encouraging innovation while maintaining safety [59]. These international models suggest that combining flexible, risk-based oversight with enhanced early engagement could help the MDR balance its safety goals with timely access and sustained innovation.

Strengths of this study include the use of both top-down and bottom-up approaches to provide a comprehensive assessment of the impact of MDR-regulated medical devices on mortality. This dual approach ensures that the estimates are robust and reliable. Additionally, the analysis is grounded in detailed data on individual medical devices, which enhances the specificity and accuracy of the findings.

Weaknesses of this analysis include inherent uncertainties in the data, particularly due to underreporting and the need to extrapolate from international evidence generated under different regulatory, reporting, and device-use conditions. Although several assumptions were chosen conservatively, they may not fully capture the complexity of the medical device market or the full spectrum of the MDR’s impact.

A further limitation concerns uncertainty in the bottom-up estimate. Formal variance estimates were not available for all device-specific inputs, so uncertainty could not be modelled probabilistically for every category. In addition, several cardiovascular and neurovascular technologies within the scope of the EU MDR were not included in the bottom-up subtotal of approximately 16,500 annual deaths avoided, which may lead to underestimation of the full mortality benefit of MDR-regulated devices.

For this reason, the bottom-up estimate was used as a device-specific plausibility check rather than as the central aggregate input. Because it fell within the independently derived top-down range of 13,000–25,000 deaths avoided annually but was likely incomplete, the midpoint of that range—19,000 deaths avoided annually—was used as the central aggregate input in downstream analyses. This choice also reduces the risk of understating access-related harms from MDR-induced market withdrawals or certification delays, because those harms depend on the mortality benefit that could be lost if access to devices is disrupted. The top-down range served as an aggregate uncertainty envelope around this central estimate.

This approach captures uncertainty in the overall mortality benefit of MDR-regulated devices, but it does not identify the contribution of individual device categories to total uncertainty. The remaining uncertainty may affect both the estimated benefits of MDR-regulated devices and, by extension, the potential harms associated with MDR-induced market disruptions.

In addition, the health benefits of MDR-regulated devices may vary substantially across clinical areas. Fields with a high prevalence of niche products, such as pediatrics or rare diseases, may face a more pronounced trade-off between improved safety and reduced availability.

Modeling the net mortality impact of the MDR over time requires further assumptions regarding the temporal distribution of deaths due to certification delays, as well as the onset and trajectory of benefits from safer devices. In particular, estimates must be made about when transitional harms peak—likely between 2023 and 2026, depending on certificate expiry profiles—and when MDR-certified products with superior safety evidence begin to reach patients. These benefits may accrue either gradually or at a constant rate, depending on market uptake and innovation cycles. These dynamics are pivotal for the timing—and, in early years, even the sign—of the net effect; central results are broadly robust across plausible time paths, but prolonged delays or concentrated withdrawals in low-substitutability niches can shift the short-term balance toward net harm.

Overall, the evidence is consistent with a net reduction in device-related mortality in Germany under central assumptions, but the estimated benefit and harm ranges substantially overlap. In the Monte Carlo analysis, the MDR scenario was associated with a net benefit in a majority of simulations, yet there remained a meaningful probability of net harm. Accordingly, conclusions should be cautious: the MDR may deliver mortality gains if the assumed safety improvements are realized, certification capacity is sustained, access disruptions are limited, and substitutes remain available, especially for niche and pediatric indications. Without these conditions, near-term harms may offset or exceed benefits. This argues for adaptive implementation, including targeted support for small-volume, high-value devices, prioritized recertification, and strong post-market action to secure long-run benefits while mitigating short-run risks.

## References

[1] European Medicines Agency. Medical Device Regulation Comes into Application. 2021. https://www.ema.europa.eu/en/news/medical-device-regulation-comes-application

[2] European Commission. Medical devices: European Commission asks for further scientific study and draws first lessons from the recent fraud on breast implants. 2012. https://ec.europa.eu/commission/presscorner/detail/en/ip_12_96

[3] Bundesinstitut für Arzneimittel und Medizinprodukte. Jahresbericht 2014/15. 2016. https://www.bfarm.de/SharedDocs/Downloads/DE/BfArM/Publikationen/Jahresbericht2014-15.pdf?__blob=publicationFile

[4] Süddeutsche Zeitung. Die Implant Files: das gefährliche Geschäft mit der Gesundheit. Süddeutsche Zeitung; 2018.

[5] Wien P, Steckeler J, Benad N. Deutsche Industrie- und Handelskammer (DIHK), MedicalMountains GmbH, & SPECTARIS. 2023.

[6] Bundesverband Medizintechnologie. EU-Kommission bessert bei MDR nach | BVMed: “Lösungen jetzt schnell umsetzen. 2023. https://www.bvmed.de/verband/presse/pressemeldungen/eu-kommission-bessert-bei-mdr-nach-bvmed-loesungen-jetzt-schnell-umsetzen

[7] Deutsches Ärzteblatt. Verband warnt: 30 Prozent aller Medizinprodukte könnten verschwinden. Deutsches Ärzteblatt; 2022.

[8] RegDesk. EU MDR transitional period extension: More time to certify medical devices. 2023. https://regdesk.co/eu-mdr-transitional-period-extension-more-time-to-certify-medical-devices/

[9] Vaupel JW, Romo VC. Decomposing change in life expectancy: a bouquet of formulas in honor of Nathan Keyfitz’s 90th birthday. Demography. 2003;40(2):201–16. doi: 10.1353/dem.2003.0018 12846129

[10] Wamble DE, Ciarametaro M, Dubois R. The effect of medical technology innovations on patient outcomes, 1990-2015: results of a physician survey. J Manag Care Spec Pharm. 2019;25(1):66–71. 29927346 10.18553/jmcp.2018.18083 PMC10398270

[11] Buxbaum JD, Chernew ME, Fendrick AM, Cutler DM. Contributions of public health, pharmaceuticals, and other medical care to US life expectancy changes, 1990-2015. Health Affairs. 2020;39(9):1546–56. 32897792 10.1377/hlthaff.2020.00284

[12] Craig A, O’Meley P, Carter P. Regulation of medical devices in Australia. EMJ Innov. 2019;3(1):56–63.

[13] International Consortium of Investigative Journalists. Implant files. 2018. https://www.icij.org/investigations/implant-files/how-medical-device-harm-is-concealed/

[14] Lalani C, Kunwar EM, Kinard M, Dhruva SS, Redberg RF. Reporting of Death in US Food and Drug Administration Medical Device Adverse Event Reports in Categories Other Than Death. JAMA Internal Medicine. 2021;181(9):1217. 10.1001/jamainternmed.2021.3942 34309624 PMC8314174

[15] Van Norman GA. Drugs and Devices: Comparison of European and U.S. Approval Processes. JACC Basic Transl Sci. 2016;1(5):399–412. doi: 10.1016/j.jacbts.2016.06.003 30167527 PMC6113412

[16] Hwang TJ, Sokolov E, Franklin JM, Kesselheim AS. Comparison of rates of safety issues and reporting of trial outcomes for medical devices approved in the European Union and United States: cohort study. BMJ. 2016;353:i3323. 10.1136/bmj.i3323 PMC4925918 27352914

[17] The Guardian. Revealed: faulty medical implants harm patients around world. 2018.

[18] OECD/European Observatory on Health Systems and Policies. Germany: Country health profile 2023. Paris: OECD Publishing. 2023.

[19] Gandjour A. A model-based estimate of the cost-effectiveness threshold in Germany. Appl Health Econ Health Policy. 2023;21(4):627–35. doi: 10.1007/s40258-023-00803-x 37039954 PMC10088581

[20] Statistisches Bundesamt. Pressemitteilung Nr. 011 vom 9. Januar 2024. 2024. https://www.destatis.de/DE/Presse/Pressemitteilungen/2024/01/PD24_011_126.html

[21] Eurostat. Healthcare resource statistics - technical resources and medical technology. 2024. https://ec.europa.eu/eurostat/statistics-explained/index.php?title=Healthcare_resource_statistics_-_technical_resources_and_medical_technology

[22] Bardy GH, Lee KL, Mark DB, Poole JE, Packer DL, Boineau R, et al. Amiodarone or an implantable cardioverter-defibrillator for congestive heart failure. N Engl J Med. 2005;352(3):225–37. 15659722 10.1056/NEJMoa043399

[23] Moss AJ, Zareba W, Hall WJ, Klein H, Wilber DJ, Cannom DS, et al. Prophylactic implantation of a defibrillator in patients with myocardial infarction and reduced ejection fraction. N Engl J Med. 2002;346(12):877–83. 11907286 10.1056/NEJMoa013474

[24] Schwab JO, Gricar B, Hauser T. Prävalenz von ICD-Patienten in Deutschland von 2010 bis 2022: Implikationen für die zukünftige Planung des Telemonitorings von Herzinsuffizienzpatienten mit ICD-Therapie. Düsseldorf: Deutsche Gesellschaft für Kardiologie – Herz- und Kreislaufforschung e. V. (DGK). 2023.

[25] Cittar M, Zecchin M, Merlo M, Piccinin F, Baggio C, Salvatore L, et al. Long-term outcomes in ICD: all-causes mortality and first appropriate intervention in ischemic and nonischemic etiologies. Am J Cardiol. 2024;233:35–44. 39370093 10.1016/j.amjcard.2024.09.026

[26] Cleland JG, Daubert JC, Erdmann E, Freemantle N, Gras D, Kappenberger L, et al. The effect of cardiac resynchronization on morbidity and mortality in heart failure. N Engl J Med. 2005;352(15):1539–49. 15753115 10.1056/NEJMoa050496

[27] Gemeinsame Bundesausschuss. Beschluss des Gemeinsamen Bundesausschusses über eine Beauftragung des IQTIG mit der Erstellung einer Weiterentwicklungsstudie für ein Qualitätssicherungsverfahren Versorgung mit implantierten Herzschrittmachern und Defibrillatoren. 2022. https://www.g-ba.de/downloads/39-261-5358/2022-03-18_IQTIG-Beauftragung_Weiterentwicklungsstudie-Herzschrittmacher-Defibrillatoren.pdf

[28] Walter N, Alt V, Rupp M. Lower limb amputation rates in Germany. Medicina (Kaunas). 2022;58(1):101. 35056409 10.3390/medicina58010101 PMC8780615

[29] Häckl D, Kossack N, Schoenfelder T. Prävalenz, Kosten der Versorgung und Formen des dialysepflichtigen chronischen Nierenversagens in Deutschland: Vergleich der Dialyseversorgung innerhalb und außerhalb stationärer Pflegeeinrichtungen. Gesundheitswesen. 2021;83(10):818–28. doi: 10.1055/a-1330-7152 33450773 PMC8497075

[30] Blankestijn PJ, Vernooij RWM, Hockham C, Strippoli GFM, Canaud B, Hegbrant J, et al. Effect of Hemodiafiltration or Hemodialysis on Mortality in Kidney Failure. N Engl J Med. 2023;389(8):700–9. 37326323 10.1056/NEJMoa2304820

[31] Kong M, Chen H, Shan K, Sheng H, Li L. Comparison of survival among adults with rectal cancer who have undergone laparoscopic vs open surgery: a meta-analysis. JAMA Netw Open. 2022;5(5):e2210861. doi: 10.1001/jamanetworkopen.2022.10861 35532937 PMC9086842

[32] Salman M, Bell T, Martin J, Bhuva K, Grim R, Ahuja V. Use, cost, complications, and mortality of robotic versus nonrobotic general surgery procedures based on a nationwide database. Am Surg. 2013;79(6):553–60. doi: 10.1177/000313481307900613 23711262

[33] Kawka M, Fong Y, Gall TMH. Laparoscopic versus robotic abdominal and pelvic surgery: a systematic review of randomised controlled trials. Surg Endosc. 2023;37(9):6672–81. doi: 10.1007/s00464-023-10275-8 37442833 PMC10462573

[34] BARMER Institut für Gesundheitssystemforschung. Hilfsmittelreport 2022. 2022. https://www.barmer.de/resource/blob/1129968/109ff5702ad14afe1579620cf3e850ef/hilfsmittelreport-2022-data.pdf

[35] Hochdruckliga. Bluthochdruck in Zahlen. https://www.hochdruckliga.de/presse/informationen/bluthochdruck-in-zahlen

[36] Satoh M, Tatsumi Y, Nakayama S, Shinohara Y, Kawazoe M, Nozato Y, et al. Self-measurement of blood pressure at home using a cuff device for change in blood pressure levels: systematic review and meta-analysis. Hypertens Res. 2025;48(2):574–91. doi: 10.1038/s41440-024-01981-4 39572787 PMC11794135

[37] Uhlig K, Balk EM, Patel K, et al. Self-Measured Blood Pressure Monitoring: Comparative Effectiveness. 45. Rockville (MD): Agency for Healthcare Research and Quality (US). 2012. https://www.ncbi.nlm.nih.gov/books/NBK84598/22439158

[38] Eggebrecht H, Mehta RH. Transcatheter aortic valve implantation (TAVI) in Germany: more than 100,000 procedures and now the standard of care for the elderly. EuroIntervention. 2019;14(15):e1549–52. doi: 10.4244/eij-d-18-01010 30530400

[39] Karagiannidis C, Krause F, Bentlage C, Wolff J, Bein T, Windisch W, et al. In-hospital mortality, comorbidities, and costs of one million mechanically ventilated patients in Germany: a nationwide observational study before, during, and after the COVID-19 pandemic. Lancet Reg Health Eur. 2024;42:100954. 39070745 10.1016/j.lanepe.2024.100954 PMC11281923

[40] Karges B, Rosenbauer J, Stahl-Pehe A, Flury M, Biester T, Tauschmann M, et al. Hybrid closed-loop insulin therapy and risk of severe hypoglycaemia and diabetic ketoacidosis in young people (aged 2-20 years) with type 1 diabetes: a population-based study. Lancet Diabetes Endocrinol. 2025;13(2):88–96. doi: 10.1016/S2213-8587(24)00284-5 39701114

[41] Fischer M, Wnent J, Gräsner JT, Seewald S, Rück L, Hoffmann H, et al. Öffentlicher Jahresbericht 2023 des Deutschen Reanimationsregisters: Außerklinische Reanimation 2023. 2024. https://www.reanimationsregister.de/berichte.htm

[42] Yan S, Gan Y, Jiang N, Wang R, Chen Y, Luo Z, et al. The global survival rate among adult out-of-hospital cardiac arrest patients who received cardiopulmonary resuscitation: a systematic review and meta-analysis. Crit Care. 2020;24(1):61. doi: 10.1186/s13054-020-2773-2 32087741 PMC7036236

[43] Heyer K, Herberger K, Protz K, Glaeske G, Augustin M. Epidemiology of chronic wounds in Germany: Analysis of statutory health insurance data. Wound Repair Regen. 2016;24(2):434–42. doi: 10.1111/wrr.12387 26609788

[44] Liu YT, Lin SH, Peng C, Huang RW, Lin CH, Hsu CC, et al. Effectiveness and safety of negative pressure wound therapy in patients with deep sternal wound infection: a systematic review and meta-analysis. Int J Surg. 2024;110(12):8107–25. 39806749 10.1097/JS9.0000000000002138 PMC11634157

[45] Institut für Qualitätssicherung und Transparenz im Gesundheitswesen. Versorgung mit implantierten Herzschrittmachern und Defibrillatoren. 2023. https://iqtig.org/downloads/berichte/2023/IQTIG_Versorgung-mit-HSM-und-DEFI_Weiterentwicklungsstudie_2023-03-31-barrierefrei.pdf

[46] Deutsche Herzstiftung. Deutscher Herzbericht – Update 2024. Frankfurt am Main: Deutsche Herzstiftung; 2024.

[47] de Koning HJ, van der Aalst CM, de Jong PA, Scholten ET, Nackaerts K, Heuvelmans MA, et al. Reduced lung-cancer mortality with volume CT screening in a randomized trial. N Engl J Med. 2020;382(6):503–13. 31995683 10.1056/NEJMoa1911793

[48] Williams MC, Wereski R, Tuck C, Adamson PD, Shah ASV, van Beek EJR, et al. Coronary CT angiography-guided management of patients with stable chest pain: 10-year outcomes from the SCOT-HEART randomised controlled trial in Scotland. Lancet. 2025;405(10475):329–37. doi: 10.1016/S0140-6736(24)02679-5 39863372

[49] Lubinski J, Kotsopoulos J, Moller P, Pal T, Eisen A, Peck L, et al. MRI surveillance and breast cancer mortality in women with BRCA1 and BRCA2 sequence variations. JAMA Oncology. 2024;10(4):493–9. 38421676 10.1001/jamaoncol.2023.6944 PMC10905376

[50] Linet MS, Slovis TL, Miller DL, Kleinerman R, Lee C, Rajaraman P, et al. Cancer risks associated with external radiation from diagnostic imaging procedures. CA Cancer J Clin. 2012;62(2):75–100. doi: 10.3322/caac.21132 22307864 PMC3548988

[51] European Commission. Rare diseases. https://health.ec.europa.eu/rare-diseases-and-european-reference-networks/rare-diseases_en#:~:text=In%20the%20EU%2C%20rare%20diseases,rare%20diseases%20start%20in%20childhood

[52] MedTech Europe. MedTech Europe Survey Report Analysing the Availability of Medical Devices in 2022 in Connection to the Medical Device Regulation (MDR) Implementation. 2022. https://www.medtecheurope.org/wp-content/uploads/2022/07/medtech-europe-survey-report-analysing-the-availability-of-medical-devices-in-2022-in-connection-to-the-medical-device-regulation-mdr-implementation.pdf

[53] European Commission. Proposal for a Regulation of the European Parliament and of the Council Amending Regulations (EU) 2017/745 and (EU) 2017/746 as Regards the Transitional Provisions for Certain Medical Devices and In Vitro Diagnostic Medical Devices. 2023. https://health.ec.europa.eu/system/files/2023-01/mdr_proposal.pdf

[54] Inxso. Core issues that terminate the MDR conformity assessment. 2025. https://inxso.pro/insights/core-issues-that-terminate-the-mdr-conformity-assessment/

[55] SimplerQMS. EU MDR Medical Device Classification: Classes, Examples. 2024. https://simplerqms.com/eu-mdr-medical-device-classification/

[56] Kadakia KT, Dhruva SS, Caraballo C, Ross JS, Krumholz HM. Use of recalled devices in new device authorizations under the US Food and Drug Administration’s 510(k) pathway and risk of subsequent recalls. JAMA. 2023;329(2):136–43. 36625810 10.1001/jama.2022.23279 PMC9857464

[57] Pharmaceuticals and Medical Devices Agency. Consultations. 2024. https://www.pmda.go.jp/english/review-services/consultations/0002.html#:~:text=PMDA%20offers%20consultations%20to%20give,on%20data%20for%20regulatory%20submissions

[58] U.S. Food and Drug Administration. Breakthrough Devices Program. 2024. https://www.fda.gov/medical-devices/how-study-and-market-your-device/breakthrough-devices-program

[59] Yadav DK, Nikraz H. Implications of evolving civil aviation safety regulations on the safety outcomes of air transport industry and airports. Aviation. 2014;18(2):94–103. doi: 10.3846/16487788.2014.926641

